# A case of minor salivary gland sialolithiasis of the upper lip

**DOI:** 10.1007/s10006-019-00745-6

**Published:** 2019-02-05

**Authors:** Atsushi Abe, Kenichi Kurita, Hiroki Hayashi, Masashi Minagawa

**Affiliations:** 1 Department of Oral and Maxillofacial Surgery, Nagoya Ekisaikai Hospital, 4-66 Syounen-cho, Nakagawa-ku, Nagoya, 454-8502 Japan; 2 Department of Oral and Maxillofacial Surgery, School of Dentistry, Aichi Gakuin University, Nagoya, Japan

**Keywords:** Minor salivary gland, Sialolithiasis, Surgical excision, Ultrasound

## Abstract

**Background:**

Sialolithiasis is the most common disease of the salivary glands. Sialolithiasis usually develops in the major salivary glands, and rarely in the minor salivary glands, with only 2% of all cases of sialolithiasis occurring in the minor salivary glands and sublingual glands. Sialoliths in the minor salivary glands result in few or no clinical symptoms and are seldom identified on imaging.

**Case presentation:**

We report herein our experience with a case of minor salivary gland sialolithiasis in a 67-year-old woman. On examination, an elastic soft, mobile, and well-circumscribed mass was palpable within the left upper lip. Ultrasound examination revealed a hypoechoic mass with heterogeneous internal echoes. The mass was excised under local anesthesia. Based on histopathological findings, a diagnosis of minor salivary gland sialolithiasis was established.

**Conclusions:**

Diagnosis of minor salivary gland sialolithiasis is challenging due to the difficulty of detecting sialoliths on imaging. A well-circumscribed mass was detected in the upper lip, and ultrasound examination revealed a round lesion, raising the suspicion of a benign tumor. Other diseases that can develop at the upper lip are calcified lymph node, phlebolith, fibroma, pleomorphic adenoma, myxoma, vascular malformation, salivary gland tumor, non-specific sialadenitis, and malignant tumor. Surgical excision is the favored approach for confirming a diagnosis of intramucosal nodular lesions.

## Background

Sialolithiasis is a common disease of the salivary glands and can be attributed to factors such as ductal obstruction, inflammation, and foreign bodies. Sialolithiasis mostly occurs in the major salivary glands, and only rarely in the minor salivary glands [1–3]. Sialoliths of the minor salivary glands typically appear as small, round, solitary submucosal nodules that are well-circumscribed and mobile. Sialolithiasis in the minor salivary glands lacks distinctive clinical features and is often invisible on radiography [4–6], complicating the diagnosis. We encountered a case of minor salivary gland sialolithiasis of the left upper lip and report our experience herein.

## Case presentation

**Fig. 1:**
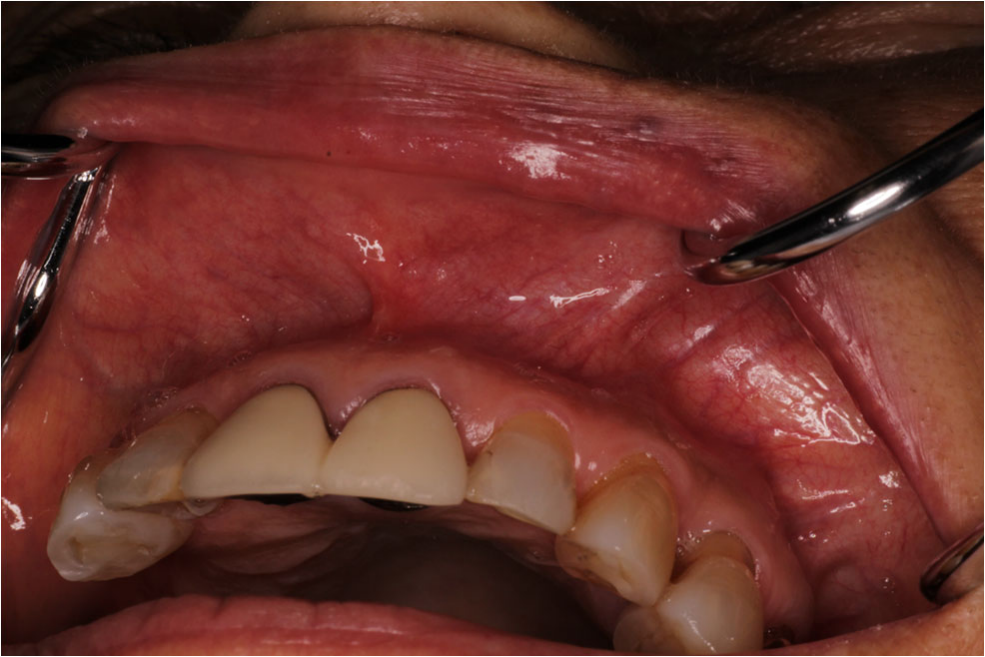
Intraoral findings. A well-circumscribed, elastic, soft, mobile, painless mass (5 mm × 5 mm) is present directly under the mucous membrane on the left side. The mucosal surface appears normal

A 67-year-old woman presented to the Department of Oral and Maxillofacial Surgery at Nagoya Ekisaikai Hospital with a chief complaint of a mass in the left upper lip. The patient had become aware of left upper lip discomfort in early August 2017, but had not sought medical attention as she was otherwise asymptomatic. She visited a local dentist for a routine checkup, who referred her to our department for further examination. In terms of medical history, she had hypertension that was adequately controlled by oral medications. Facial appearance was symmetrical, with no swelling of the lips (Fig. 1). On intraoral examination, an elastic soft mass measuring 5 mm × 5 mm was palpable within the left upper lip. The mass was mobile and well-circumscribed. The overlying mucosal surface was normal in color, with no evidence of ulceration.

**Fig. 2:**
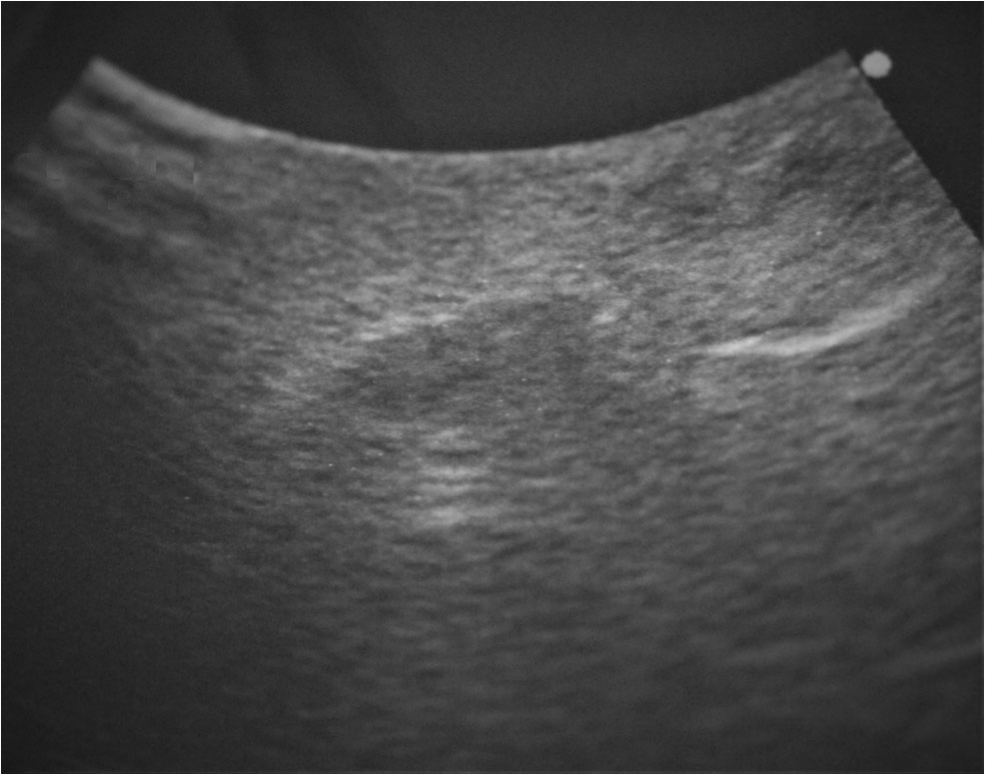
Ultrasound findings. Ultrasonography reveals a hypoechoic mass (5 mm × 5 mm × 5 mm) with heterogeneous internal echoes

**Fig. 3:**
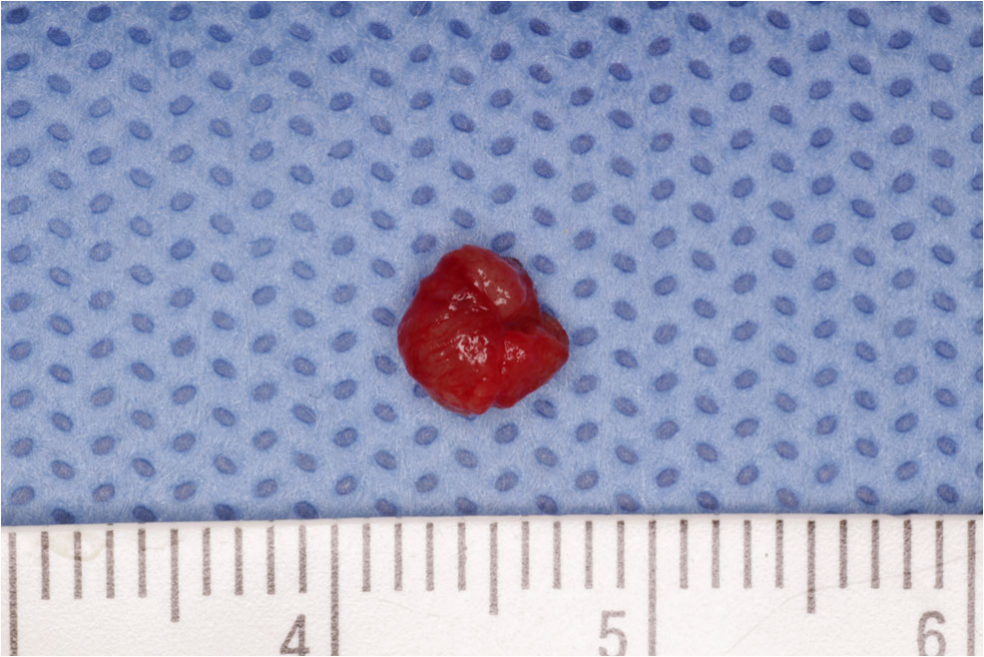
Excised specimen. The mass is solid and round with a smooth, dark-red surface

**Fig. 4:**
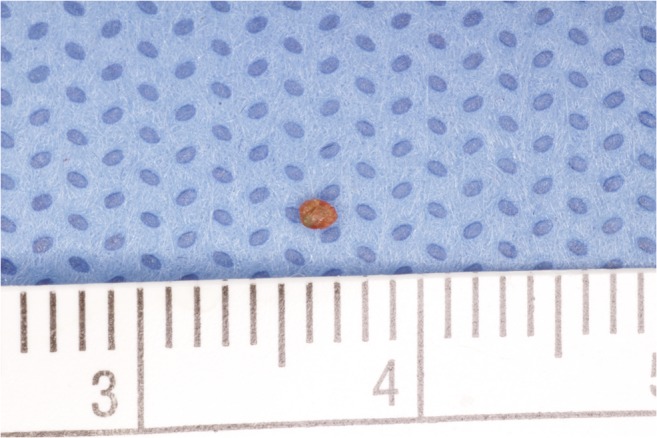
Sialolith. The size of the sialolith is 2 mm × 2 mm × 2 mm

Ultrasound examination revealed a hypoechoic mass (5 mm × 5 mm × 5 mm) with heterogeneous internal echoes, but without calcification (Fig. 2). The provisional diagnosis was benign tumor of the left upper lip. The mass and overlying mucosa were excised under local anesthesia. The mass was solid and round with a smooth, dark-red surface (Figs. 3 and 4). The excision was straightforward, with no adhesion to the surrounding tissue. No invasion into muscle was noted. The postoperative course was uneventful without infection and lip movement was good. To date, 1 year postoperatively, the patient has experienced no recurrence of the disease.

**Fig. 5:**
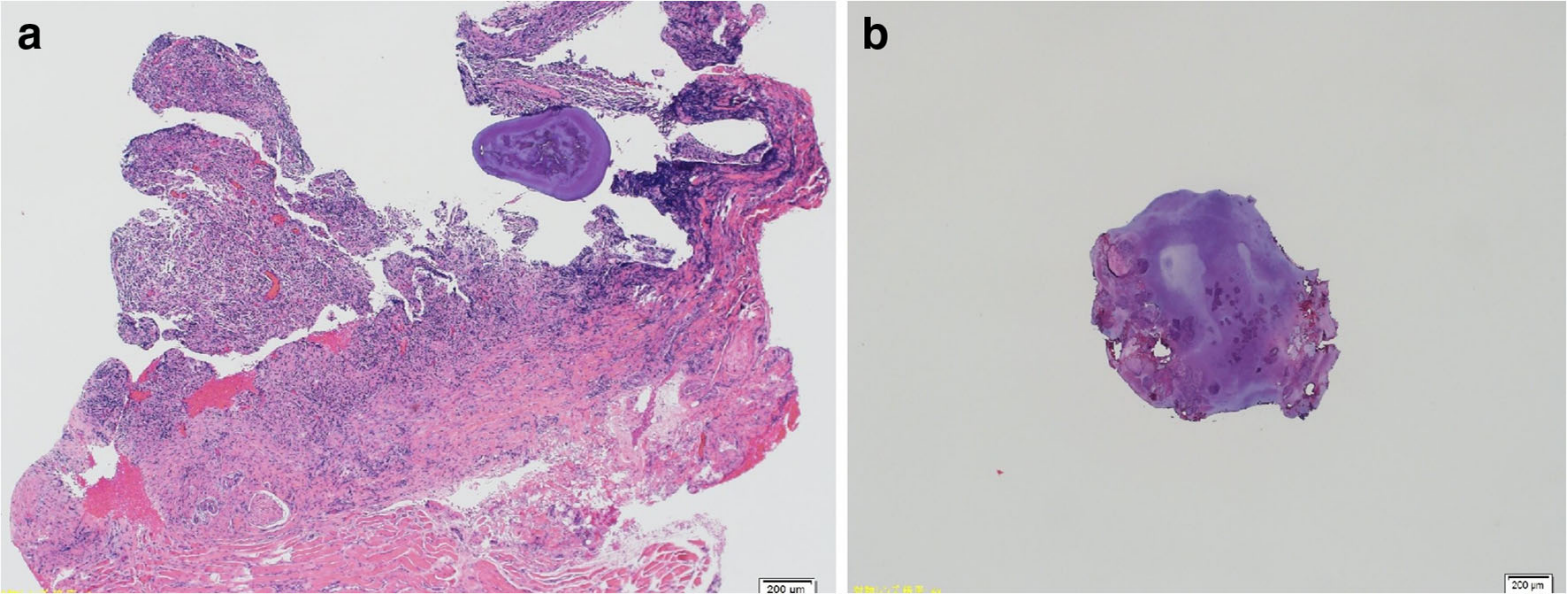
Histopathological findings. **a** A mildly dilated salivary gland duct is surrounded by granulation tissue and fibrous connective tissue with inflammatory cell filtration. **b** The sialolith comprises amorphous substance of various densities and granular material of various sizes

Histopathological examination showed a mildly dilated salivary duct surrounded by granulation tissue and fibrous connective tissue with infiltration of inflammatory cells (Fig. 5). No evidence of tumor was noted. The sialolith comprised an amorphous substance of varying density and granular material of various sizes. Based on these findings, a diagnosis of minor salivary gland sialolithiasis was established.

## Discussion

Sialolithiasis is a condition in which a sialolith forms within a salivary gland or excretory duct and represents the most frequent disease of the salivary glands. Sialoliths form most often in the submandibular glands (92%), followed by the parotid glands (6%). The incidence is low for minor salivary glands and the sublingual glands: 2% for both sites combined [7].

There are at least two potential reasons for the infrequent occurrence of sialoliths in minor salivary glands [6]. First, sialoliths that develop in minor salivary glands can be as large as 5 mm, but most are around 1–2 mm [6, 8, 9]. Because salivary calculi of the minor salivary gland are small, they are only rarely discovered on imaging. A second reason may be that secretion from the major salivary glands is controlled by nerves, whereas that from the minor salivary glands is spontaneous and not mediated by nervous stimulation. Salivary stasis is thus unlikely in the minor salivary glands, presumably lowering the risk of sialolith development [10].

Although the precise etiology of sialolith formation is unknown, two distinct phases are thought to be involved [7–9]. During the first phase, salivary stasis and spasmodic contractions of the ducts are induced by irritant factors, such as changes in the chemical properties of saliva, accumulation of saliva, foreign bodies, bacterial infection (fungi and actinomycetes), inflammation, and microlith formation in a salivary gland, as well as saliva acidosis or hypoptyalism. During the second phase, deposition of calcium salts leads to the formation of a calculus. Sialolithiasis thus often develops in older adults, who are prone to regressive changes in the salivary glands, acinar atrophy, and formation of microliths in the salivary glands [4]. As the patient in the present case was a 67-year-old woman, these factors were likely to have contributed to sialolith formation.

Patients with minor salivary gland sialolithiasis typically present with painless swelling or mass formation and lack specific clinical symptoms [1–3]. The condition is therefore usually asymptomatic with no salivary colic, and hard masses are mostly impalpable. However, in rare cases, a fistula may form in association with sialolith formation [5]. A well-circumscribed elastic soft mass was detected in the present case, and ultrasound examination revealed a round lesion, raising the suspicion of a benign tumor. Other diseases that can develop at the same site are calcified lymph node, phlebolith, fibroma, pleomorphic adenoma, myxoma, vascular malformation, salivary gland tumor, non-specific sialadenitis, and malignant tumor [6, 9, 11]. Minor salivary gland sialolithiasis is thus considered to be one of the diseases requiring differential diagnosis as a mass-forming condition occurring in the region of the salivary glands.

Histopathological findings of minor salivary gland sialolithiasis include a dilated duct in minor salivary gland tissue, acinar atrophy, and periductal inflammation. These findings are essential for distinguishing the disease from mucus-secreting, low-grade mucoepidermoid carcinoma [8]. A long-standing sialolith often exhibits a laminar structure surrounding a central core of homogeneous amorphous matter, while a more recent sialolith often entirely comprises homogeneous amorphous matter [11].

The sialolith in the present case was small and somewhat amorphous, and was thus considered relatively new. In addition, the lesion showed only inflammatory granulation tissue and fibrous connective tissue with inflammatory cell infiltration, ruling out a diagnosis of neoplastic disease. Sialolithiasis in a minor salivary gland is difficult to diagnose accurately based on clinical findings alone and clinical and histopathological diagnoses show poor consistency [4, 8, 9]. This is due to the extremely small sizes of the lesions, making sialoliths hard to detect on computed tomography or ultrasonography. Some studies have reported that the use of low-dose intraoral radiography may be effective in detecting sialoliths [7]. However, this modality is not fully reliable and therefore impractical. If diagnostic imaging fails to detect a sialolith in a minor salivary gland, surgical excision is the favored approach for confirming the diagnosis [7–9]. In the present case, the lesion was too small for needle biopsy, and the lesion was difficult to evaluate presurgically using magnetic resonance imaging or contrast-enhanced computed tomography. We thus performed ultrasonography, and the resulting findings led to a provisional diagnosis of benign tumor. If excisional biopsy demonstrates the lesion to be benign, no further treatment is necessary. However, the possibility of malignant tumor should also be considered before surgery, as additional treatment would then be required.

## Conclusions

Diagnosis of sialolithiasis in minor salivary glands is challenging, and the number of cases diagnosed may underestimate the true incidence of the disease. Because salivary calculi of the minor salivary gland are small, they are only rarely discovered on imaging. As detecting a sialolith via imaging is difficult, surgical excision is the best approach to confirm the diagnosis of intramucosal nodular lesions.

## Data Availability

All data used for this report are included in the text.

## References

[1] Rallis G, Mourouzis C, Zachariades N (2004). A study of 55 submandibular salivary gland excisions. Gen Dent.

[2] Lustmann J, Regev E, Melamed Y (1990). Sialolithiasis. A survey on 245 patients and a review of the literature. Int J Oral Maxillofac Surg.

[3] Lustmann J, Shteyer A (1981). Salivary calculi: ultrastructural morphology and bacterial etiology. J Dent Res.

[4] Anneroth G, Hansen LS (1983). Minor salivary gland calculi. A clinical and histopathological study of 49 cases. Int J Oral Surg.

[5] Souza IF, Kawatake MM, Soares AB, Moraes PC, Araujo VCD, Passador-Santos F (2015). Sialolithiasis of minor salivary glands. Rev Gaúch Odontol.

[6] Lee LT, Wong YK (2010). Pathogenesis and diverse histologic findings of sialolithiasis in minor salivary glands. J Oral Maxillofac Surg.

[7] Ben Lagha N, Alantar A, Samson J, Chapireau D, Maman L (2005). Lithiasis of minor salivary glands: current data. Oral Surg Oral Med Oral Pathol Oral Radiol Endod.

[8] Brazao-Silva MT, Prosdocimi FC, Lemos-Junior CA, de Sousa SO (2015). Clinicopathological aspects of 25 cases of sialolithiasis of minor salivary glands. Gen Dent.

[9] Wang W-C, Chen C-Y, Hsu H-J, Kuo J-H, Lin L-M, Chen Y-K (2016). Sialolithiasis of minor salivary glands: a review of 17 cases. J Dent Sci.

[10] Harrison JD. Histology and pathology of sialolithiasis. In Witt RL, eds; Salivary gland diseases: surgical and medical management. 1st edition, Thieme medical Publishers, New York, 2006, p71–8

[11] Kasaboglu O, Er N, Tumer C, Akkocaoglu M (2004). Micromorphology of sialoliths in submandibular salivary gland: a scanning electron microscope and x-ray diffraction analysis. J Oral Maxillofac Surg.

